# Complete plastid genome of *Gentiana trichotoma* (Gentianaceae) and phylogenetic analysis

**DOI:** 10.1080/23802359.2019.1644231

**Published:** 2019-07-30

**Authors:** Shanshan Sun, Hui Wang, Pengcheng Fu

**Affiliations:** School of Life Science, Luoyang Normal University, Luoyang, People’s Republic of China

**Keywords:** *Gentiana trichotoma*, phylogenetic analysis, plastome

## Abstract

The complete plastid genome of *Gentiana trichotoma* was determined and analyzed in this work. It had a circular-mapping molecular with the length of 144,759 bp, has similar gene composition with *G.* section *Cruciata* but contains 10 more genes than *G.* section *Kudoa*. Phylogenetic analysis showed that *G. trichotoma* clustered together with section *Kudoa* rather than section *Cruciata*. The plastome provided in this work would be useful for elucidation of *Gentiana* evolution.

As a big genus containing 15 sections (Ho and Liu 2001), *Gentiana* plants are typically alpine and important parts of alpine shrub and meadow. *Gentiana trichotoma* Kusnezow, belonging to section *Frigidae* Kusnezow, is endemic to the Qinghai-Tibetan Plateau (Ho and Liu 2001). However, there has been no genomic studies in section *Frigidae*.

Herein, we reported and characterized the complete *G. trichotoma* platome (MN089577). One *G. trichotoma* individual (specimen number: Fu2016163-6) was collected from Daocheng, Sichuan Province of China (29°27′09″N, 100°13′27″E) and its voucher specimens was deposited in the herbarium of School of Life Science, Luoyang Normal University. The fragmented genomic DNA was sequenced using Illumina HiSeq 2500 platform (Novogene, Tianjing, China), yielding approximately 5 Gb of 150-bp paired-end. The plastome was *de novo* assembled in NOVOPlasty 2.6.1 (Dierckxsens et al. 2016) and then annotated in GeSeq (Tillich et al. 2017) using the default parameters. Comparative analysis was conducted in mVISTA (Frazer et al. 2004) with *G. straminea* (Ni et al. 2016) and *G. lawrencei* var. *farreri* (Fu et al. 2016) which represents the only two plastome-available *Gentiana* sections, *Cruciata* Gaudin, and *Kudoa* (Masamune) Satake & Toyokuni ex Toyokuni, respectively. Shared protein-coding genes in plastomes of available *Gentiana* species were extracted and concatenated, then aligned using MAFFT (Katoh et al. 2002). The ML phylogeny was performed with IQ-TREE (Nguyen et al. 2015) in PhyloSuite (Zhang et al. 2018) with 1000 replicates. *Swertia mussotii* (KC875852) and *S. verticillifolia* (MF795137) were served as the outgroups.

The complete *G. trichotoma* platome is a circular-mapping molecule with the length of 144,759 bp. The LSC, IR, and SSC regions were 77,430, 25,162, and 17,005 bp, respectively. The overall GC content of the platome was 37.8%. A total of 130 genes were annotated, containing 88 protein-coding genes, 34 tRNA genes, and 8 rRNA genes. Comparison analysis indicated that platome of *G. trichotoma* has similar gene composition with section *Cruciata* (Ni et al. 2016; Zhou et al. 2018), with hotspots locating at intergenic regions such as *trnK-^UUU^*–*rps16*, *atpH*–*atpI*, *petN*–*trnD,* and *trnL-^UAG^*–*ccsA*. However, gene loss was not detected in *G. trichotoma*, which is different with section *Kudoa* which has almost lost 10 *ndh* genes (Sun et al. 2018). Phylogenetic analysis showed that *G. trichotoma* was clustered together with section *Kudoa* rather than section *Cruciata* (Figure 1), which is consistent with the previous study (Favre et al. 2016). *Gentiana stipitata* belonging to section *Kudoa* was clustered with section *Cruciata*, which is consistent with the previous study (Sun et al. 2018), indicating that more taxa should be involved for further study. The determination of the *G. trichotoma* platome sequences provided new molecular data to illuminate the *Gentiana* evolution.

**Figure 1.**
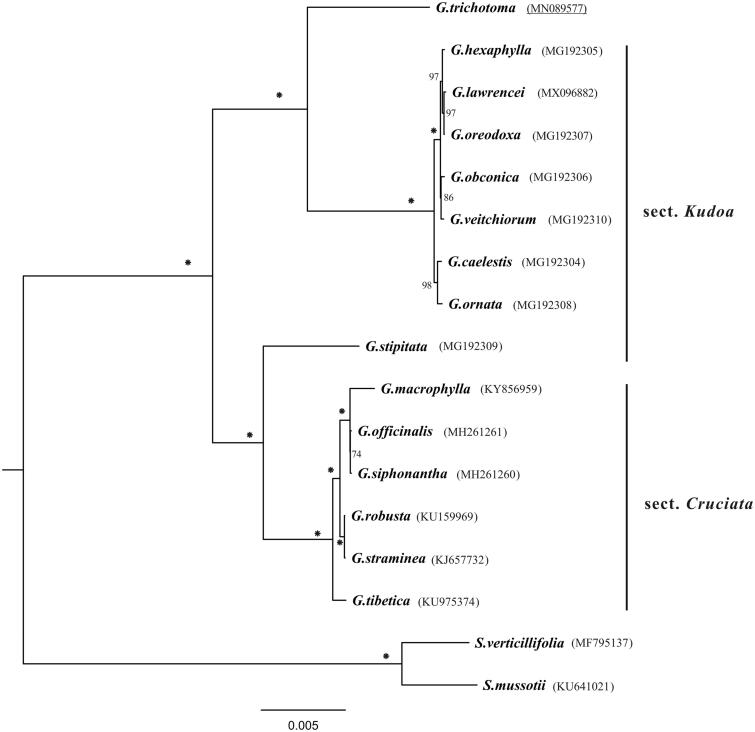
Phylogenetic tree (maximum likelihood) based on protein-coding genes of *Gentiana* plastomes. The asterisks along the branches mean 100% bootstrap supports based on 1000 replicates. The underline located in Genbank accession numbers indicates newly determined plastid genomes.
